# The association between chronic kidney disease and tuberculosis; a comparative cohort study in England

**DOI:** 10.1186/s12882-020-02065-4

**Published:** 2020-10-01

**Authors:** Judith Ruzangi, Masao Iwagami, Liam Smeeth, Punam Mangtani, Dorothea Nitsch

**Affiliations:** 1 Department of Non-Communicable Disease Epidemiology, London School of Hygiene and Tropical Medicine, London, UK; 2 Department of Health Services Research, Faculty of Medicine, University of Tsukuba, building #861, 1-1-1 Tenno-dai, Tsukuba, Ibaraki, Japan; 3 Department of Infectious Disease Epidemiology, London School of Hygiene and Tropical Medicine, London, UK

**Keywords:** Tuberculosis, Chronic kidney disease, Epidemiology, Primary care, CPRD

## Abstract

**Background:**

People with end-stage kidney disease have an increased risk of active tuberculosis (TB). Previous systematic reviews have demonstrated that patients with chronic kidney disease (CKD) have increased risk of severe community-acquired infections. We investigated the association between CKD (prior to renal replacement therapy) and incidence of TB in UK General Practice.

**Methods:**

Using the UK Clinical Practice Research Datalink, 242,349 patients with CKD (stages 3-5) (estimated glomerular filtration rate < 60 mL/min/1.73 m^2^ for ≥3 months) between April 2004 and March 2014 were identified and individually matched (by age, gender, general practice and calendar time) to a control from the general population without known CKD. The association between CKD (overall and by stage) and incident TB was investigated using a Poisson regression analysis adjusted for age, gender, ethnicity, socio-economic status, chronic obstructive pulmonary disease (COPD) and diabetes.

**Results:**

The incidence of TB was higher amongst patients with CKD compared to those without CKD: 14.63 and 9.89 cases per 100,000 person-years. After adjusting for age, gender, ethnicity, socio-economic status, diabetes and COPD, the association between CKD and TB remained (adjusted rate ratio [RR] 1.42, 95% confidence interval [CI] 1.01–1.85). The association may be stronger amongst those from non-white ethnic minorities (adjusted RR 2.83, 95%CI 1.32–6.03, *p*-value for interaction with ethnicity = 0.061). Amongst those with CKD stages 3–5, there was no evidence of a trend with CKD severity.

**Conclusions:**

CKD is associated with an increased risk of TB diagnosis in a UK General Practice cohort. This group of patients should be considered for testing and treating for latent TB.

## Background

Chronic kidney disease (CKD) is recognised as a growing public health problem [1]. CKD stages 3–5 (defined as estimated glomerular filtration rate [eGFR] < 60 mL/min/1.73 m^2^ for ≥3 months) are estimated to affect approximately 6% of the general population in England [2]. CKD can progress to end-stage kidney disease (ESKD) requiring renal replacement therapy (RRT) in a small but substantial proportion of people [3]. CKD is associated with a range of comorbidities and increased risk of infection and is classed as an independent risk factor for active tuberculosis (TB) [3–6].

Tuberculosis (TB) disease is a global public health problem with imperfect control options [7]. In England, despite a low TB burden setting and a reduction in overall TB cases in recent years, the proportion of TB cases with at least one risk factor (homelessness, drug or alcohol misuse or imprisonment) has increased in the last year [8]. The burden of disease is concentrated in large urban areas with London having the highest TB incidence of any western European capital [8, 9]. There is evidence that a large number of new TB diagnoses in the UK come from activation of latent TB [9]. The recent decline of overall TB incidence in England has been attributed partly to the impact of testing and treating patients with latent TB, the UK TB pre-entry screening programme and improvements in TB control [8]. Efforts to further identify, access, screen and treat those at high risk of TB are in order to achieve year on year reductions in TB. A systematic review in 2015 found evidence of an increased risk of active TB in patients with ESKD compared to the general population [10]. However, none of these studies were based in the UK nor adjusted for comorbidities or lifestyle factors. Specifically, the review was unable to explore the influence of diabetes and other known TB risk factors with the data and so could not assess the extent of confounding by these. A recent cohort study in a population in Taiwan suggested an increased risk of TB in early stages of CKD [11]. However, the background incidence of TB in this population is far higher than that of the UK.

We used a large representative primary care database to compare the rates of incident TB in those with and without CKD stages 3–5, excluding those on RRT and assessed the dose-response relationship between CKD stages and TB incidence. We also explored whether the CKD-TB association varied with age and ethnic group.

## Methods

### Data sources

In the UK, the primary care system acts as a gatekeeper to healthcare-patients (excluding those in prisons, military service and the homeless) need to be registered with a primary care doctor to access National Health Service (NHS) nonemergency care. The Clinical Practice Research Datalink (CPRD) is an ongoing primary care database of routinely recorded primary care electronic data. Diseases can be identified using diagnostic Read codes in the data. Over 650 general practices contributed data meeting quality control standards to the CPRD, covering and representing (with respect to age, sex and ethnicity) nearly 7% of the UK population [12]. We used CPRD linked to the inpatient Hospital Episodes Statistics (HES) for more accurate and complete information of ethnicity [13] which is available for approximately 75% of participating general practices in England. Data were also linked to the Office of National statistics (ONS) data for mortality information and to the Index of Multiple Deprivation (IMD) in quintiles as assigned by postcode of residence [14]. Ethical approval for this study was obtained from the Independent Scientific Advisory Committee (ISAC), which oversees research involving de-identified CPRD data (Protocol 17_124R) as well as from the LSHTM Research Ethics Committee (reference: 14162).

### Study population

All living adults (> 18 years) contributing to HES-linked CPRD anytime from 1 April 2004 to 31 March 2014 were eligible for inclusion. We excluded patients already receiving RRT (haemodialysis, peritoneal dialysis, and kidney transplantation) prior to cohort entry (Additional file 1). Patients were eligible for inclusion at the latest of: the date that the practice reached CPRD quality control standards or 1 year after practice registration [15] or 1 April 2004 (testing for CKD was incentivised in 2004 for those with diabetes and in 2006 for those at risk of CKD). Follow-up continued until the incidence of TB diagnosis, death, incidence of RRT, change of practice, last data collection from the practice or 31/03/2014 (end date of HES version 10 linkage eligibility).

### Definition of exposure and outcome

During the study period, CKD was classified into 5 stages using eGFR and markers of kidney damage. Markers of kidney damage include a urinary albumin: creatinine ratio greater than 3 mg/mmol, urine sediment abnormalities and others are listed in the National Institute of Care and Excellence (NICE) Guidelines [3]. CKD stages 3–5 status was defined in patients based on two consecutive measurements of eGFR < 60 mL/min/1.73m^2^ taken more than 3 months apart [16]. Patients, including those who had CKD stages 3–5 before April 2004, were included in the cohort on the date when they first satisfied the CKD stages 3–5 definition (i.e. second eGFR < 60 ml/min/1.73m^2^) during the eligibility period as previously defined [17]. As a comparison group, patients without known CKD stages 3–5 (with or without creatinine measurement) were selected randomly from the same practice population, matched on age, sex, and calendar time (i.e. index date). Identification and management of CKD and other chronic diseases are expected to depend on GP and calendar time [18, 19]. Each control entered the cohort on the same date as their CKD stages 3–5 counterpart. Controls who developed CKD stages 3–5 (i.e. second eGFR < 60 ml/min/1.73m^2^ more than 3 months after the first measure) later were censored at the time of satisfying CKD definition and contributed separately as an incident patient with CKD stages 3–5 from that point forward with their own matched control. In a subgroup analysis amongst those with CKD, patients were classified with different stage of CKD at baseline: CKD stage 3a (eGFR 45–59 mL/min/1.73m^2^), CKD stage 3b (eGFR 30–44 mL/min/1.73m^2^), and CKD stage 4 or 5 (eGFR < 30 mL/min/1.73m^2^).

**Fig. 1:**
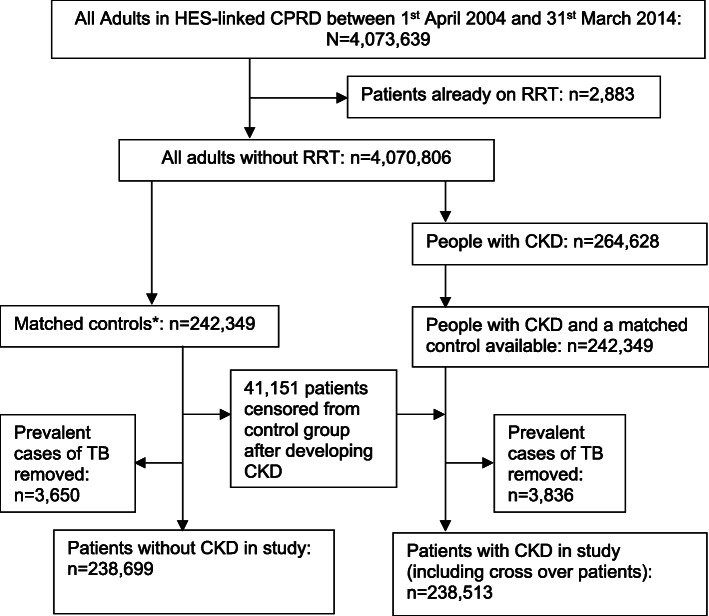
Flow chart of the selection of matched patients with and without chronic kidney disease stages 3–5. CKD = chronic kidney disease stages 3–5, CPRD = clinical practice research datalink, HES = hospital episode statistics, RRT = renal placement therapy. *Matched control randomly selected individuals without CKD. 41,151 patients censored from control group after developing CKD. They were included in CKD cohort from that point forward

TB diagnosis was based on a list of TB diagnosis codes as developed in a previous study [20]. We excluded patients with a history of TB diagnosis at cohort entry (Fig. 1). Incident TB was defined as a recorded TB diagnosis for the first time in CPRD after the cohort entry (i.e. the date of satisfying CKD definition for CKD stages 3–5 patients and the same date for matched controls). By assuming that GPs record a previous TB diagnosis before GP registration within a certain period of time after GP registration (1 year), the first recorded diagnosis (after 1 year from GP registration) of TB is regarded as a new TB experience in his/her life.

### Covariates

People at risk of TB infection and disease are those who are close contacts with an infectious TB case, immunocompromised, healthcare workers, without a BCG vaccination, live in/come from a high TB prevalent country, are very young or old and from underserved groups. Those with silicosis, on chemotherapy, with excessive alcohol use, or those with diabetes may be more likely to develop TB [6]. We used ethnicity, age, IMD as a proxy for socio-economic status, gender, body mass index (BMI), smoking and chronic illnesses such as diabetes, cancer and chronic obstructive pulmonary disease (COPD) and rheumatoid arthritis to approximate/measure these factors [21–25]. Risk factors associated with CKD progression include, cardiovascular disease, age, proteinuria, acute kidney injury, hypertension, diabetes, smoking, African, African-Caribbean or Asian family origin, chronic use of nonsteroidal anti-inflammatory drugs (NSAIDs) and untreated urinary outflow tract obstruction [26]. Hypertension was considered to be on the causal pathway as it is also an outcome of worse kidney function [27]. Previous literature shows that low SES, high BMI, persistent asthma, cancer, COPD and rheumatoid arthritis are risk factors for CKD [28–33]. Factors that were risk factors for TB and were associated with CKD were treated as potential confounders.

Based on previous studies using UK primary care data, we classified patients with no record of ethnicity as white [34, 35]. Age and financial year were analysed as categorical variables, with groups < 55, 55–64, 65–74, 75–84,> = 85 and from 1 April to 31 March for every 2 years respectively. Chronic diseases were recorded as binary variables (i.e. presence or absence of each condition at the time of cohort entry). Socio-economic status was allocated at an individual-level by quintile using 2010 ONS estimates of the IMD (composite area-level marker of deprivation) [36]. For patients with missing individual-level social economic status, we used the social economic status for the patient’s general practice. Patient body mass index (BMI) (kg/m^2^) was grouped into BMI < 18.5 as underweight, 18.5-25.0 as normal weight, 25.0-30 as overweight and ≤ 30 as obese. Smoking status was recorded as current smokers, ex-smokers and non-smoker in the patient record. Both BMI and smoking status were taken from data recorded closest to index date. Therefore, it was assumed that these states had not changed during the study period. See Additional file 1 for further methodology details on variable construction.

### Statistical analysis

**Fig. 2:**
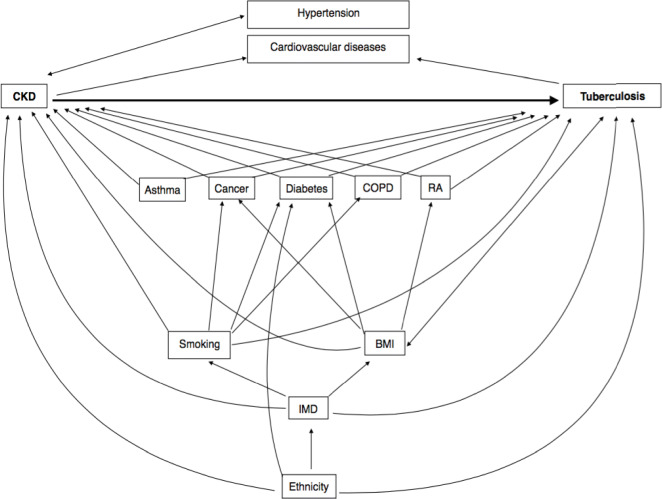
Conceptual diagram of associations between chronic kidney disease stages 3–5 and tuberculosis. BMI = body mass index, IMD = index of multiple deprivation, RA = Rheumatoid arthritis, CKD = chronic kidney disease stages 3–5

All data were analysed using Stata 14 software (Stata Corp, Texas). Baseline characteristics among patients with and without CKD were compared using χ^2^ tests. Throughout this study we used unconditional Poisson regression analysis to investigate the association between CKD status and TB incidence taking account of clustering by general practice using random errors. We did not use matched analysis as we removed prevalent TB cases so the original matching was not maintained, but analysis was adjusted for matching factors (age, gender, calendar year, random effect for general practice). Using a forward analysis, models were developed with guidance from the relationships between variables on a conceptual diagram since not all potential confounders could be adjusted for in the final model as there were only 246 TB cases (Fig. 2) (Additional file 2). Variables were kept in the model if they changed the point estimate for rate ratio of TB in those with CKD. With the addition of each variable, we checked for multicollinearity by analysing changes in standard errors and point estimates. We assessed the influence of BMI on results and found that addition of this variable led to an increase in estimates, whilst addition of diabetes led to attenuation of association. Hence, to have a conservative estimate, in the final model we adjusted for age, gender, ethnicity, IMD, diabetes and COPD. We used likelihood ratio methods to test whether CKD is associated with TB diagnosis after adjusting for these six factors.

### Subgroup analysis

In examining whether there was a dose-response relationship between CKD (stage 3a, 3b, and 4) and TB incidence, we included CKD stage as a continuous score variable in the models.

### Effect modification

A priori effect modification of the relationship between CKD and TB by age and ethnicity was explored by using likelihood ratio tests. We were concerned that the age modification was driven by non-whites since age and ethnicity are closely correlated as a result of past migration patterns, so we explored the effect modification with age in whites only. We tested for interaction after adjusting for gender, IMD and diabetes.

## Results

As shown in Fig. 1, among 4,070,806 eligible patients (median age 39 [IQR 27–56], female 51.2%), 264,628 had CKD (median age 77 [IQR 71–83], male 38.7%). Of these, 242,349 patients were matched by age, gender, general practice and cohort entry date to a control without CKD. Unmatched patients with CKD (*n* = 22,279) were more likely to be female and older (median age 88 [IQR 84–92], female 68.5%). Of the 242,349 matched control patients, 41,151 (17.0%) were later found to have CKD (therefore, were included in the CKD cohort from that time point forward and are included in the final CKD cohort of 238,513). After excluding existing TB cases at index date (7486 patients), 477,212 patients were left with follow-up time from almost a month to 10 years (median 3.81 years).

Patients with CKD were more likely to be overweight with BMI ≥ 25 kg/m^2^, deprived and ex-smokers compared to those without CKD. Chronic illnesses were more common in those with CKD compared to those without CKD (Table 1). Those who were South Asian, male, underweight, most deprived, diabetic or asthmatic were more frequently diagnosed with TB. Those with COPD, cancer and rheumatoid arthritis showed increased rates compared to patients without these factors (Additional file 2). There was strong evidence of within-GP practice clustering of TB cases (likelihood ratio test for no clustering (θ = 0); *P* < 0.05) throughout the analysis which was accounted for by the statistical model.

**Table 1:** Baseline characteristics of matched patients with and without chronic kidney disease

	Patients without CKD *N =* 238,699	Patients with CKD *N =* 238,513	*p*-value
	Number (%)	Number (%)	
Age (years)			
< 55	6823 (2.9)	6785 (2.8)	0.990
55–64	23,377 (9.8)	23,278 (9.8)	
65–74	70,027 (29.3)	69,963 (29.3)	
75–84	100,761 (42.2)	100,733 (42.3)	
> =85	37,711 (15.8)	37,754 (15.8)	
Gender			
Male	93,860 (39.3)	93,824 (39.3)	0.912
Female	144,839 (60.7)	144,689 (60.7)	
Ethnicity			
White/not-recorded^a^	233,154 (97.7)	233,179 (97.8)	
South Asian	1712 (0.7)	2153 (0.9)	< 0.001
Black	1144 (0.5)	1041 (0.4)	
Other^b^	2689 (1.1)	2140 (0.9)	
Index of Multiple Deprivation^c^			
1(least deprived)	55,982 (23.5)	52,223 (21.9)	
2	60,777 (25.5)	59,575 (25.0)	
3	49,736 (20.8)	49,931 (20.9)	< 0.001
4	41,539 (17.4)	43,986 (18.4)	
5(most deprived)	30,665 (12.9)	32,798 (13.8)	
Smoking status			
Non-Smoker	91,030 (38.1)	79,432 (33.3)	
Current Smoker	35,834 (15.0)	28,796 (12.1)	< 0.001
Ex-Smoker	105,966 (44.4)	129,414 (54.3)	
Missing	5869 (2.5)	871 (0.4)	
Body mass index (kg/m^2^)			
< 18.5	6469 (2.7)	4454 (1.9)	
18.5–25	83,966 (35.2)	68,760 (28.8)	< 0.001
25–30	79,336 (33.2)	86,724 (36.4)	
≥ 30	39,854 (16.7)	62,385 (26.2)	
Missing	29,074 (12.2)	16,190 (6.8)	
Chronic physical illnesses			
Asthma	27,389 (11.5)	30,595 (12.8)	< 0.001
Cancer	46,603 (19.5)	53,450 (22.4)	< 0.001
COPD	14,544 (6.1)	17,739 (7.4)	< 0.001
Diabetes	23,986 (10.1)	52,141 (21.9)	< 0.001
Rheumatoid arthritis	4189 (1.8)	5922 (2.5)	< 0.001

*CKD* chronic kidney disease stages 3–5

*COPD* chronic obstructive pulmonary disease

^a^white/not recorded: 136119 (56.2%) and 140,784 (58.1%) patients with and without CKD stages 3–5, respectively, had missing ethnicity

^b^other: Mixed, not stated and other ethnicities

^c^Index of Multiple Deprivation*: 259 (0.1%) and 272 (0.1%) patients with and without CKD stages 3–5 were missing individual data so* Index of Multiple Deprivation *of general practice was used*

The overall incidence rate of TB in those without CKD, and in those with CKD was 9.89 (7.96–12.30) and 14.63 (12.28–17.42) per 100,000-person years, respectively. After controlling for age, gender, ethnicity, socio-economic status, COPD and diabetes, there was strong evidence for a positive association between CKD status and TB incidence (adjusted rate ratio [RR] = 1.42, 95%CI 1.09–1.85, *p* = 0.008) (Table 2). No multicollinearity was observed.

**Table 2:** Incidence of tuberculosis diagnosis by chronic kidney disease status

	No/ person years^b^	Rate^c^ (95%CI)	Unadjusted rate ratio (95%CI)	Adjusted rate^a^ ratio (95%CI)
Patients without CKD (*N* = 238,699)	92/946306.71	9.89 (7.96–12.30)	1 (reference)	1 (reference)
Patients with CKD (*N =* 238,513)	154/1068622.53	14.63 (12.28–17.42)	1.48 (1.14–1.91)	1.42 (1.09–1.85)

*CI* confidence intervals, *CKD* chronic kidney disease stages 3–5

^a^adjusted for age, gender, ethnicity, socio-economic status, chronic obstructive pulmonary disease and diabetes

^b^No. of patients diagnosed with tuberculosis / total follow up time in person years

^c^*Per 100,000 person-years*

### Subgroup analysis

After controlling for age, gender, IMD, ethnicity, COPD and diabetes, the rate ratio of TB incidence for an increase in one unit in CKD stage amongst those with CKD stages 3–5 is 1.09 (95%CI 0.82–1.46). There was no evidence of a trend with CKD severity (*p* = 0.541).

### Effect modification

There was borderline evidence that ethnicity modified the effect of CKD (*p* = 0.061) even after adjusting for age, gender, IMD, COPD and diabetes (Table 3), suggesting that the rate of incident TB associated with CKD is much higher in those of non-white descent. On crude analyses there was an interaction with age, however, this interaction may have been driven by the fact that incident TB cases occurred in younger people of non-white ethnicity. Hence, we explored the interaction with age amongst whites only. Whilst the point estimates suggest a trend with those of younger age being more susceptible, the confidence intervals are wide, and the result may be due to chance (Table 4).

**Table 3:** Association of chronic kidney disease with incident tuberculosis by ethnic group

Group	Number of patients	Tuberculosis cases/PY^a^	Adjusted rate ratio^c^ (95%CI)	*P*-value^b^	Test for interaction *p*-value
White/not recorded^d^	466,350	210/1970832.9	1.35 (1.02–1.78)	0.036	0.061
Non-White	10,862	36/44096.337	2.83 (1.32–6.03)	0.007	

*CKD* chronic kidney disease stages 3–5. *PY* person years

^a^total follow up time

^b^*p*-value: from Wald test

^c^for age, gender, socio-economic status, COPD and diabetes

^d^white/not recorded: 136119(56.2%) and 140,784 (58.1%) patients with and without CKD, respectively, had missing ethnicity

**Table 4:** Association of chronic kidney disease with tuberculosis by age in those who are White only

Age (years)	Adjusted rate ratio^**a**^ (95%CI)	Test for interaction ***p***-value
< 55	6.67 (0.80–55.51)	*p* = 0.137
55–64	3.26 (1.06–10.00)	
65–74	1.27 (0.81–1.97)	
75–84	1.21 (0.80–1.85)	
> = 85	0.81 (0.29–2.32)	

*CKD* chronic kidney disease stages 3–5

^a^adjusted for gender, socio-economic status and diabetes

## Discussion

### Key findings and interpretation

In this large study, patients with known CKD were more likely to be diagnosed with TB than patients without CKD. There was no evidence that the rate of incident TB diagnosis varied with severity of kidney function. The association of CKD with TB may vary by ethnicity with a stronger association seen in those of non-white ethnicity.

The results of a positive association between CKD and incident TB agree with the systematic review reporting an increased risk of acute community-acquired infections in adult patients with CKD in high-income countries [4]. Authors have previously hypothesised that as kidney function declines the immunity of the host could be reduced through various mechanisms related to gradual accumulation of uraemic metabolites. It was possible that the risk of TB associated with CKD only manifested after a long period, so this study may have underestimated long-term risks. The reported median period of follow up in this study was 3.81 years. The effect of potential confounders on the effect estimate of CKD on incident TB overall was small and did not appreciably affect estimates of association. Previous studies on the risk of active TB in ESKD patients have not adjusted for socio-economic status, lifestyle factors, or chronic illnesses such as diabetes and COPD [37, 38]. Having found this association there are a number of open questions that need to be investigated in future research using more granular data. It may be that CKD affects the severity of TB manifestation in those with latent TB. The association of TB with CKD may be a combination of increased risk of infection and risk of disease as a result of decreased immunity in these patients.

The lack of evidence for the variation of TB diagnosis rate with severity of kidney function may have been driven by competing risk of death since death was a marker for end of follow up. A previous study showed evidence of a graded association between reduced eGFR and the risk of death, cardiovascular events, and hospitalization [39]. We did not adjust for competing risk of death as there was lack of power in groups with severe CKD given that there were so few TB cases overall.

There was borderline evidence that effect of CKD on TB varied with ethnicity. This interaction has to be confirmed in a larger, diverse cohort study as 97.7% of the present study were white or have no recorded ethnicity (assumed white). Those who were of non-white ethnicity experienced a greater CKD effect than whites. This could be due to higher proportions of latent TB cases present in non-whites than whites at the start of the cohort. The findings were however consistent with a previous study in South-East London which documented that approximately 2/3 of cases with TB in the renal unit were amongst people from non-UK born ethnic minorities [40].

### Strengths and limitations

To our knowledge this is the largest cohort study to date investigating the association between CKD before ESKD and incidence of TB with individual-level adjustments for patient demographic and comorbidities. The finding of increased TB risk in people with CKD found in this study agrees with a similar published study and reviews looking at different infections in comparable study populations, and TB for studies comparing patients on dialysis to the general population [4, 10, 11]. We used a detailed source of routine data which is representative of the UK population thereby minimising selection bias [12].

There are a number of limitations pertaining to the source of the dataset. There was potential of non-differential misclassification of TB. Currently, latent TB is tested in those at risk of having TB. There is no current guideline recommending screening of pre-dialysis CKD patients for latent TB and hence awareness of risk of disease so bias would tend towards diluting the association. We assumed that TB patients would seek medical care in a setting as the UK where access to healthcare is free. Diagnosis of TB in secondary care is highly likely to be communicated to the GP for public health purposes and due to high risk of side effects from anti-TB medications as well as the interactions of such medicines with others prescribed by GP.

Serum creatinine testing in primary care is recommended for people who are considered to be at risk for CKD [3, 17]. Patients with unmeasured CKD may have been misclassified as a control, which could underestimate the true CKD-TB association. However, the prevalence of CKD identified in CPRD has been shown to be similar to that estimated in a nationally-representative survey (Health Survey for England), hence most CKD patients as defined by reduced eGFR are probably captured in CPRD [41]. Defining CKD using eGFR from creatinine measurements is more accurate than using diagnoses in primary care databases [42].

CKD is a complication of HIV [43]. HIV infection and/or treatment for the infection or complications may be associated with development of CKD. HIV infection is a well-known a risk factor for developing TB [6, 44]. If HIV was a confounder in this cohort, the effect estimate calculated may be overestimated. We did not identify nor adjust for HIV status as this is underreported in GP records [45]. We did not adjust for BCG vaccination status. Infant BCG vaccination is recommended for those at high risk in the UK. This immunity wanes with time so may or may not offer little protection from TB for the majority of this cohort because of their age [46].

We assumed smoking status, IMD, BMI and chronic illness status taken on/close to index date stayed constant throughout the follow-up period. This would likely result in residual confounding from non-differential misclassification of these confounders.

There may be a risk of reverse causality especially if the CKD and TB diagnoses are close together, however, in this cohort TB of the kidney was rarely recorded. Similarly, there may be reverse causality between BMI and TB diagnosis, potentially biasing the true association between CKD and TB diagnosis.

## Conclusion

We find evidence for an increased risk of TB amongst those with CKD not requiring RRT in a UK General Practice cohort. This study is beneficial to UK policy seeking to identify high risk groups in primary care to test and treat for latent TB to prevent disease– based on the present study presence of CKD stages 3–5 is associated with risk of incident TB. These real-world data highlight useful further research investigating the association between CKD and TB in patients of non-white ethnicity.

## Supplementary information

**Additional file 1: Table A1.** List of medcodes for Renal Replacement Therapy definition. Further Details on Methodology.

**Additional file 2: Table A2.** Univariate associations between potential confounders, financial year and incident tuberculosis rate. **Table A3.** Effect of chronic kidney disease on rate of tuberculosis adjusted for potential confounders.

## Data Availability

Data are available on request from the CPRD. Their provision requires the purchase of a license, and our license does not permit us to make them publicly available to all. We used data from the version collected in February 2017 and have clearly specified the data selected in our Methods section. To allow identical data to be obtained by others, via the purchase of a license, we will provide the code lists on request. Licences are available from the CPRD (http://www.cprd.com): The Clinical Practice Research Datalink Group, The Medicines and Healthcare products Regulatory Agency, 5th Floor, 151 Buckingham Palace Road, Victoria, London SW1 W 9SZ.

## Abbreviations

CKD: Chronic kidney disease
IMD: Index of Multiple Deprivation
ONS: Office of National Statistics
HES: Hospital Episodes Statistics
NHS: National Health Service
CPRD: Clinical Practice Research Datalink
eGFR: Estimated glomerular filtration rate
ESKD: End-stage kidney disease
RRT: Renal replacement therapy
ISAC: Independent Scientific Advisory Committee
LSHTM: London School of Hygiene and Tropical Medicine
NSAIDs: Nonsteroidal anti-inflammatory drugs
