# Supplementary material for: The association between chronic kidney disease and tuberculosis; a comparative cohort study in England
Source: BMC Nephrol. 2020 Oct 1;21:420. doi: 10.1186/s12882-020-02065-4 (PMC7528250; doi:10.1186/s12882-020-02065-4)
Supplement: Supplementary file 1 — Additional file 1: Table A1. List of medcodes for Renal Replacement Therapy definition. Further Details on Methodology. [file 12882_2020_2065_MOESM1_ESM.docx]

**Table A1 List of medcodes for Renal Replacement Therapy (RRT) definition**

| medcode | Read term |
| --- | --- |
| 2994 | Peritoneal dialysis |
| 2996 | Haemodialysis NEC |
| 2997 | Transplantation of kidney |
| 5504 | Transplantation of kidney NOS |
| 5911 | [V]Kidney transplanted |
| 8037 | Insertion of ambulatory peritoneal dialysis catheter |
| 11553 | Kidney transplant failure and rejection |
| 11745 | Transplantation of kidney from live donor |
| 11773 | Dialysis for renal failure |
| 17253 | Renal transplant planned |
| 18774 | Renal transplant with complication, without blame |
| 20073 | Renal dialysis |
| 20196 | H/O: renal dialysis |
| 22252 | [V]Renal dialysis status |
| 23773 | Removal of ambulatory peritoneal dialysis catheter |
| 24361 | Transplantation of kidney from cadaver |
| 26862 | Exploration of renal transplant |
| 28158 | Kidney dialysis with complication, without blame |
| 30709 | Insertion of temporary peritoneal dialysis catheter |
| 30756 | Continuous ambulatory peritoneal dialysis |
| 31549 | Compensation for renal failure |
| 36442 | Placement ambulatory dialysis apparatus - compens renal fail |
| 44422 | H/O: kidney dialysis |
| 46145 | [V]Aftercare involving renal dialysis NOS |
| 48022 | Other specified compensation for renal failure |
| 48057 | Renal tubulo-interstitial disordrs in transplant reject |
| 49028 | H/O: kidney recipient |
| 54844 | [X]Failure sterile precautions dur kidney dialys/other perf |
| 54990 | Kidney transplant with complication, without blame |
| 56760 | Placement ambulatory apparatus compensation renal failure |
| 59194 | Placement ambulatory apparatus- compensate renal failure OS |
| 60302 | Creation of graft fistula for dialysis |
| 60743 | [V]Aftercare involving intermittent dialysis |
| 64636 | Compensation for renal failure NOS |
| 64828 | Peritoneal dialysis NEC |
| 65089 | Placement other apparatus- compensate for renal failure NOS |
| 66705 | Allotransplantation of kidney from live donor |
| 66714 | Renal dialysis with complication, without blame |
| 69266 | Failure of sterile precautions during kidney dialysis |
| 69427 | Accid cut,puncture,perf,h'ge - perfusion NOS |
| 69760 | [X]Other dialysis |
| 70712 | Det.ren.func.after ren.transpl |
| 70874 | Other specified transplantation of kidney |
| 71124 | Haemofiltration |
| 72004 | Excision of rejected transplanted kidney |
| 83513 | Placement other apparatus for compensation for renal failure |
| 88597 | Automated peritoneal dialysis |
| 89924 | Allotransplantation of kidney from cadaver, heart-beating |
| 90952 | Pre-transplantation of kidney work-up, recipient |
| 93366 | Interventions associated with transplantation of kidney |
| 94964 | Post-transplantation of kidney examination, live donor |
| 96095 | Pre-transplantation of kidney work-up, live donor |
| 96133 | Allotransplantation kidney from cadaver, heart non-beating |
| 96184 | Accid cut,puncture,perf,h'ge - kidney dialysis |
| 98364 | Allotransplantation of kidney from cadaver |
| 100693 | [X]Renal tubulo-interstitial disorders/transplant rejection |
| 101756 | Thomas intravascular shunt for dialysis |

**Further details on methodology**

**Categorisation of Variables**

Age was analysed as a categorical variable with groups <55, 55-64, 65-74, 75-84 and >=85 according to World Health Organisation provisional guidelines on standard international age classification.

We adjusted for financial year because the rate of tuberculosis varied with time during 2004-2014 and primary care financial incentives (e.g. the Quality outcomes framework) and the data collection are by financial year. Included financial year as a categorical variable, from 1 April to 31 March for every 2 years.

Chronic diseases were recorded as binary variables (i.e. presence or absence of each condition at the time of cohort entry).

Socio-economic status (SES) was allocated at an individual-level by quintile using 2010 Office of National Statistics (ONS) estimates of the Index of Multiple Deprivation (IMD) (composite area-level marker of deprivation) (1). For patients with missing individual-level social economic status, we used the social economic status for the patient’s general practice.

The numbers of Non-white ethnicities were small and some of the ethnic groups were similar, example Caribbean, African and Other Black are all Black. To reduce type II error, we re-categorised the ethnicity variable provided. Those who were British, Irish and Other White were classed as White, those who were White and Black Caribbean, White and Black African, White and Asian and Other Mixed were classed as Mixed, those who were Indian, Pakistani, Bangladeshi and Other Asian were classed as South Asian, those who were Caribbean, African and Other Black were classed as Black whereas Chinese was classed with other ethnic groups as Other. Additionally, there were those who had not stated their ethnic group and those that were missing a value.

Based on previous studies using UK primary care data, we classified patients with missing ethnicity values as white (2,3). This strategy was effective in our matched cohort with median age of 76 years old, because older people are more likely to be white according to the UK census. Therefore, ethnicity was grouped into 4 groups, White (including missing), South Asian and Black as above. The fourth group included those who were mixed, had not stated and other ethnic groups. These were classed as Other.

For the interaction analysis, ethnicity was categorised into White and Non-white populations to provide sufficient TB events in each stratum.

Patient body mass index (BMI) (kg/m^2^) was categorised according to the World Health Organisation classification. BMI<18.5 was underweight, 18.5<BMI<25.0 as normal weight, 25.0≤BMI<30 as overweight and ≤30 as obese.

Smoking status was recorded as current smokers, ex-smokers and non-smoker. Both BMI and smoking status was taken from data recorded closest to index date. Therefore, it was assumed that these states had not changed during the study period.

**Sample size considerations**

The rate ratio between patients with and without CKD stages 3-5 was expected to be at least 1.5 according to a previous study (4). Setting α error as 5% and power as 80%, we require at least 120 and 80 incident cases of TB. Therefore, the result from the feasibility count, 246 incident cases with TB (154 CKD patients and 92 controls), was considered sufficient for this analysis. We used the conceptual diagram below as guidance to formulate the models in a forward analysis since not all potential confounders could be adjusted for in the final model based on 246 TB cases.

**Footnotes**

SES-socio-economic status

ONS-Office of National Statistics

IMD-index of multiple deprivation

BMI-body mass index

CKD-chronic kidney disease

**References**

1. English indices of deprivation 2015 - GOV.UK [Internet]. [cited 2017 Sep 10]. Available from: https://www.gov.uk/government/statistics/english-indices-of-deprivation-2015

2. Hippisley-Cox J. Predicting risk of upper gastrointestinal bleed and intracranial bleed with anticoagulants: Cohort study to derive and validate the QBleed scores. BMJ. 2014;349.

3. Hippisley-Cox J, Coupland C. Derivation and validation of updated QFracture algorithm to predict risk of osteoporotic fracture in primary care in the United Kingdom: Prospective open cohort study. BMJ. 2012;345(7864).

4. Al-Efraij K, Mota L, Lunny C, Schachter M, Cook V, Johnston J. Risk of active tuberculosis in chronic kidney disease: a systematic review and meta-analysis. Int J Tuberc Lung Dis. 2015;19(12):1493–9.
