# Supplementary material for: The association between chronic kidney disease and tuberculosis; a comparative cohort study in England
Source: BMC Nephrol. 2020 Oct 1;21:420. doi: 10.1186/s12882-020-02065-4 (PMC7528250; doi:10.1186/s12882-020-02065-4)
Supplement: Supplementary file 2 — Additional file 2: Table A2. Univariate associations between potential confounders, financial year and incident tuberculosis rate. Table A3. Effect of chronic kidney disease on rate of tuberculosis adjusted for potential confounders. [file 12882_2020_2065_MOESM2_ESM.docx]

**Table A2 Univariate associations between potential confounders, financial year and incident TB rate**

| Variable | Unadjusted Rate Ratio (95%CI) | Likelihood ratio test P-value |
| --- | --- | --- |
| Age(years) |  |  |
| <55 | 1 |  |
| 55-64 | 0.75 (0.35-1.61) |  |
| 65-74 | 0.96 (0.48-1.90) |  |
| 75-84 | 0.87 (0.44-1.71) | 0.214 |
| >=85 | 0.54 (0.24-1.22) |  |
|  |  |  |
| Gender |  |  |
| Male | 1 |  |
| Female | 0.61 (0.47-0.78) | <0.001 |
|  |  |  |
| Ethnicity |  |  |
| White/not-recorded* | 1 |  |
| South Asian | 16.62 (11.07-24.96) |  |
| Black | 2.98 (0.94-9.43) | <0.001 |
| Other** | 1.90 (0.70-5.14) |  |
|  |  |  |
| Social Economic Status |  |  |
| 1(least deprived) | 1 |  |
| 2 | 0.96 (0.64-1.44) |  |
| 3 | 1.40 (0.95-2.08) | 0.058 |
| 4 | 1.31 (0.86-1.98) |  |
| 5(most deprived) | 1.65 (1.07-2.54) |  |
|  |  |  |
| Smoking Status |  |  |
| Non-smoker | 1 |  |
| Current Smoker | 1.17 (0.81-2.54) | 0.503 |
| Ex-smoker | 0.94 (0.71-1.24) |  |
|  |  |  |
| Body Mass Index (kg/m^2^) |  |  |
| <18.5 | 1 |  |
| 18.5-25 | 0.51 (0.28-0.96) |  |
| 25-30 | 0.35 (0.19-0.65) | 0.001 |
| >=30 | 0.33 (0.17-0.64) |  |
|  |  |  |
| Chronic illnesses |  |  |
| Diabetes | 1.41 (1.03-1.92) | 0.036 |
| Asthma | 2.71 (2.04-3.59) | <0.001 |
| Cancer | 1.34 (1.00-1.80) | 0.060 |
| COPD | 4.07 (2.95-5.61) | <0.001 |
| Rheumatoid Arthritis | 2.10 (1.12-3.96) | 0.039 |
|  |  |  |
| Financial year |  |  |
| 1/04/2004 - 31/03/2006 | 1 |  |
| 1/04/2006 - 31/03/2008 | 1.02 (0.74-1.40) |  |
| 1/04/2008 - 31/03/2010 | 1.51 (1.06-2.14) | 0.282 |
| 1/04/2010 - 31/03/2012 | 1.11 (0.63-1.96) |  |
| 1/04/2012 - 31/03/2014 | 1.03 (0.38-2.80) |  |

CKD-chronic kidney disease stages 3-5

*white/not recorded: 136119(56.2%) and 140784(58.1%) patients with and without CKD stages 3-5, respectively, had missing ethnicity

Social Economic Status: 259 (0.1%) and 272(0.1%) patients with and without CKD stages 3-5 were missing individual data so social economic status of general practice was used

COPD=chronic obstructive pulmonary disease

**other=mixed, not stated and other ethnicities

*

**Table A3 Effect of chronic kidney disease on rate of tuberculosis (TB) adjusted for potential confounders**

|  | Rate Ratio (95%CI) | P-value* | P-value** |
| --- | --- | --- | --- |
| No CKD | 1 (reference) |  | 0.008 |
| CKD | 1.48 (1.14-1.91) | 0.003 |  |
|  |  |  |  |
| Factors adjusted for |  |  |  |
| Financial year | 1.49 (1.15-1.92) | 0.003 |  |
| Age | 1.49 (1.15-1.92) | 0.003 |  |
| Gender | 1.49 (1.15-1.93) | 0.002 |  |
| Ethnicity | 1.46 (1.13-1.89) | 0.004 |  |
| Social Economic Status | 1.47 (1.13-1.90) | 0.004 |  |
| Smoking | 1.48 (1.14-1.92) | 0.003 |  |
|  |  |  |  |
| Diabetes | 1.43 (1.10-1.85) | 0.008 |  |
| Asthma | 1.45 (1.12-1.88) | 0.005 |  |
| Cancer | 1.47 (1.13-1.90) | 0.004 |  |
| COPD | 1.44 (1.11-1.86) | 0.006 |  |
| Rheumatoid Arthritis | 1.47 (1.13-1.90) | 0.004 |  |
|  |  |  |  |
| Model 1 | 1.46 (1.12-1.89) | 0.004 |  |
| Model 2 | 1.37 (1.05-1.78) | 0.019 |  |
| Model 3 | 1.39 (1.07-1.81) | 0.014 |  |
|  |  |  |  |
| Final model | 1.42 (1.09-1.85) | 0.008 |  |

CKD-chronic kidney disease stages 3-5

COPD- chronic obstructive pulmonary disease

Model 1: Age, gender, ethnicity and socio-economic status

Model 2: Diabetes, asthma, chronic obstructive pulmonary disease, cancer, and rheumatoid arthritis

Model 3: Diabetes and chronic obstructive pulmonary disease

Final model adjusted for age, gender, ethnicity, socio-economic status, chronic obstructive pulmonary disease (COPD) and diabetes

*P-value from Wald test

**P-value from likelihood ratio test
